# Editorial for Special Issue “Biotechnology for Environmental Remediation”

**DOI:** 10.3390/microorganisms14020283

**Published:** 2026-01-26

**Authors:** Elena Efremenko

**Affiliations:** Faculty of Chemistry, Lomonosov Moscow State University, Lenin Hills 1/3, Moscow 119991, Russia; elena_efremenko@list.ru

Environmental pollution continues to be an urgent global problem that requires innovative solution approaches, and biotechnology promises to address environmental problems and also serve as a scientific and practical basis for the remediation of various natural and artificial systems. Due to the current state of affairs and future market forecasts [1], biotechnological tools possess immense potential for the effective removal of pollutants, the mitigation of the effects of pollution, and the restoration of ecosystems. Various types of pollutants (e.g., petroleum products, metals, pesticides, pharmaceuticals, toxins, hormones, alkaloids, ionic liquids, detergents, microplastics, prions, etc.) and their complex combinations are the main targets of the microbiological purification of waters, soils, and the atmosphere. Advanced methods, whether already known or newly emerging, for the effective treatment and degradation of accumulated industrial and agricultural wastes also represent areas of significant interest for basic and applied biotechnology.

The main objects of research in the field of remediation biotechnology include various strains of microorganisms and their enzymes, natural and artificial microbial consortia, biotechnological processes using active biocatalysts, and carriers of microbial cells. Within this framework, combinations of certain biocatalytic systems in multi-stage processes are also attractive components of microbiological processes developed for environmental bioremediation. All new achievements in this field are based on contemporary accumulated scientific knowledge, which makes it possible to choose specific microorganisms and conditions for the treatment of a specific substrate (pollutant).

This editorial introduces this Special Issue of *Microorganisms* on “Biotechnology for Environmental Remediation”, which comprises 13 articles that highlight the progress and importance of new approaches to the remediation of different environments using newly isolated or process-adapted microorganisms that can demonstrate improved beneficial functions compared to known analogues. The participation of authors from 12 countries (China, Russia, South Africa, Spain, Chile, Greece, Egypt, Bangladesh, Peru, the Republic of Korea, Japan, and the USA) in the preparation of these review and research articles confirms the internationality of the biotechnological and environmental remediation problems discussed. The content of the performed investigations reflects the multi- and interdisciplinary nature of research activity in this field, where various microorganisms (bacteria, microalgae, and fungi) are at the forefront of the solutions being sought.

This Special Issue aimed to stimulate the dissemination of new achievements and research results to specialists working across a range of fields to address environmental pollution by deploying biotechnological remediation. Relying on the latest achievements and breakthroughs in this field, the authors of the Special Issue sought to inspire readers to embark on new innovative journeys and fresh applications of cutting-edge technologies in the interests of people from all countries.

As the Special Issue was being conceived, key research areas were identified, which seemed to be the most relevant activity at the time, and enabled us to achieve good fit with the scope of *Microorganisms*. The selected areas of interest encompassed the following topics:

- Biotechnology and biocatalysts for water, air, and soil purification—the application of biotechnological approaches to soil and wastewater treatment, including those based on microbial enzymes;
- Biodegradation and bioremediation with a focus on the role of microbial enzymes, microorganisms, and microbiological processes in the decomposition and detoxification of various pollutants;
- Nanobiotechnological innovations inspired by biological systems for environmentally friendly and sustainable recovery strategies;
- Innovative methods for controlling and predicting metabolic cell activity, and identifying and discriminating microbial living cells in biotechnological processes focused on environmental remediation;
- Combinations of various microorganisms for the treatment of pollutants with the study of the accumulation and conversion of pollutants from water and soil.

The authors presented works in accordance with the stated goals of this Special Issue, fitting the development of their research to the specified thematic areas. At the same time, all of the pulished works, including both reviews and experimental studies, stood out in terms of the originality, novelty, and practical significance of the data provided.

Despite their diversity, all of the articles in this Special Issue can be roughly divided into two main blocks. The first consists of a number of works devoted to tackling the negative impacts of heavy metals (Contributions 1–4), as well as arsenic (Contribution 5), in aquatic and soil environments. The authors of these studies found that there are both bacterial and fungal cultures capable of demonstrating high resistance to the presence of chromium, cadmium, lead, zinc, cobalt, and arsenic.

The strains of microorganisms identified by the authors appeared capable not only of accumulating biomass in the presence of these pollutants, but also of participating in processes such as metal reduction, the stabilization of insoluble inorganic and organic phosphorus, and the biosynthesis of siderophores and indole-3-acetic acid, which promote plant growth, including of plants growing in areas with metal-contaminated soils. The authors established that the additional use of biochar (Contribution 3), which can be obtained from the processing of animal wastes [2], as well as additional phytoremediation (Contribution 4), contribute to the treatment of highly polluted wastewater, particularly water resulting from leather production, and large-scale soil restoration at mining sites. The demonstrated results provide economically feasible and environmentally sustainable solutions based on the potential of the applied microorganisms.

The achievements of Contribution 1, which focuses on the production of “green nanobioparticles”, is worthy of special mention. These nanobioparticles are formed in the living cell biomass of microorganisms, most often different types of fungi. The metals typically form complex compounds with microbial biomolecules, which can then be successfully used for catalysis of reactions in environmentally oriented processes [3]. The authors showed a unique result, this being that palladium nanoparticles formed with the participation of hyphae of the fungus Neurospora crassa proved to be promising catalysts for the complete reduction of Cr(VI) within a few minutes.

The second block of articles in this Special Issue is devoted to the degradation of various wastewater and soil pollutants. In some investigations, in which individual microbial cultures were used, metabolic activity was identified by the authors when necessary (Contributions 6–8). In other works, greater attention was paid to the application of different consortia of microorganisms to solving bioremediation problems and analyzing their effectiveness under the influence of various factors (Contributions 9–13). Overall, the studies presented demonstrate the ability of bacteria, including cyanobacteria, to degrade pollutants not only in aquatic environments (Contributions 6, 9, and 11) but also in soil (Contribution 3), confirming not only the processes of pollutant removal but also their conversion into commercially significant products (for example, cheese whey is converted into polyhydroxyalkanoates in Contribution 6, and caffeine-reduced extracts from coffee grounds are used for bioelectricity generation in Contribution 11).

Of the articles in this Special Issue, the one that attracts particular interest is Contribution 7, in which the authors search for active hydrocarbon destructors of crude oil and diesel fuel among microorganisms sourced from agricultural soils. Traditionally, similar searches for destructors have been carried out in places of existing hydrocarbon pollution with the expectation of detecting cells adapted to such soil contamination. However, the authors of this study followed the path of searching for the most active hydrocarbon destructors in fertile soils, and this unusual step proved successful, since soil fertility is determined not only by the ratio of carbon to nitrogen, but also by the presence of active enzymes produced by the microorganisms present [4].

It should be noted that the authors of the studies presented in this Special Issue are particularly interested in consortia of microorganisms involved in the biotechnological degradation of various wastes and pollutants, including substances of natural origin such as lignin (Contribution 10) and xenobiotics (pesticides, pharmaceuticals, plastic materials, polycyclic aromatic hydrocarbons, etc., as in Contribution 10). It appears that various fungal cells play notable roles in such consortia due to their various extracellular enzymes (Contributions 11 and 13). These complex microbial systems of heterogeneous composition, combining prokaryotes and eukaryotes capable of jointly participating in various bioremediation processes, attract the attention of researchers by enabling them to accumulate knowledge on the general patterns of the existence of such complex biosystems (Contributions 6 and 10–13), to artificially create natural-like consortia (Contribution 13), and to manage them via biotechnological processes aimed at solving various bioremediation tasks.

One of the reviews in this Special Issue (Contribution 12) draws attention to water pollutants that are not yet subject to environmental regulatory control, but that have been shown to have adverse effects on human health and ecosystems. These substances include agricultural chemicals, industrial compounds, or by-products of industrial processes, personal care products, pharmaceuticals, secondary products, nanomaterials, and their metabolites. These compounds can enter the environment through agricultural, laboratory, domestic, and hospital wastewater, as well as industrial effluents, street effluents, atmospheric precipitation, etc.

The main problem with these substances is their dose, which can lead to pronounced negative effects not only in the aquatic environments themselves, but also in soils, where such substances can bioaccumulate and become bio-enhanced, passing their toxic effects on to microorganisms and other living organisms. This can in turn lead to a partial change in the composition of microorganisms, resulting in a further decrease in the efficiency of the decomposition of such pollutants in soil systems.

The assessment of negative consequences of pollutants and the search for ways to overcome them are important and can be carried out using accessible databases containing information on the annotated genomes of microorganisms that are present in the target environments, primarily in soils. This is the subject of one of the articles in this Special Issue (Contribution 8), in which the authors use cells of the genus Rhodococcus to analyze their known genomic composition and determine the practical potential of individual representatives of these widespread soil bacteria to possibly achieve the biocontrol and bioremediation of environments contaminated with antibiotics, pesticides, and other xenobiotics.

This article once again proves that the analysis of large databases in the search for new potential participants in bioremediation processes is a modern trend that could be called “bioremediation in silico” [5]. This kind of “dry” research makes it possible to analyze theoretical data and make predictions that can later be tested in practice.

It can be unequivocally said that the main research areas of the articles presented in this Special Issue will remain relevant and attractive to readers in the near future, since they correspond to the main modern trends in the field. Thus, according to current publications, interest in the bioremediation of media contaminated with metals [6], petroleum hydrocarbons [7], different pharmaceuticals [8], including antibiotics [9], and various synthetic pollutants [10] is still strong. In the forthcoming decade, an important role in these processes will clearly be assigned not only to bacterial and microalgae cells [11], but also to microscopic fungi [12], since knowledge about them is constantly accumulating. In this regard, the number of studies and interested researchers exploring the possibility of using microbial consortia of various compositions [13] to solve complex problems in bioremediation biotechnology will certainly not decrease.

In all likelihood, development of technological solutions enabling simulation of the environmental processes using both natural and artificial microbial consortia will be even more intensive in the near future [14]. At the same time, the selection of candidates for such processes and the possible construction of artificial consortia will continue to be carried out using genomic analysis that takes into account microbial diversity, new revealing taxa, and metabolic pathways [15]. Such approaches have previously been mentioned in relation to the use of genomic databases.

It is clear that the long-standing interest in microbial fuel cells, in which various microorganisms can participate in the generation of bioelectric energy through the conversion of organic pollutants concurrently with bioremediation of aquatic environments, is unlikely to decrease—only the pollutants under study are likely to change [16].

Thus, the articles presented in the Special Issue are closely related to the mainstream of modern research in the field of bioremediation and cover all of the field’s main directions.

## Data Availability

Not applicable.

## List of Contributions

1. Lai, H.; Tan, L.; Shi, Z.; Huang, S.; Yu, W.; Wei, G.; Xie, J.; Zhou, S.; Tian, C. Enhancing the catalytic performance of PdNPs for Cr (VI) reduction by increasing Pd (0) content. *Microorganisms* **2025**, *13*, 1346. https://doi.org/10.3390/microorganisms13061346.
2. Tuli, S.R.; Ali, M.F.; Jamal, T.B.; Khan, M.A.S.; Fatima, N.; Ahmed, I.; Khatun, M.; Sharmin, S.A. Characterization and molecular insights of a chromium-reducing bacterium *Bacillus tropicus*. *Microorganisms* **2024**, *12*, 2633. https://doi.org/10.3390/microorganisms12122633.
3. Zhu, G.; Li, Y.; Cheng, D.; Chen, R.; Wang, Y.; Tu, Q. Effects of distiller’s grains biochar and *Lactobacillus plantarum* on the remediation of Cd-Pb-Zn-contaminated soil and growth of sorghum-sudangrass. *Microorganisms* **2024**, *12*, 2592. https://doi.org/10.3390/microorganisms12122592.
4. Bi, B.; Xiao, Y.; Xu, X.; Chen, Q.; Li, H.; Zhao, Z.; Li, T. Diversity and functional roles of root-associated endophytic fungi in two dominant pioneer trees reclaimed from a metal mine slag heap in southwest China. *Microorganisms* **2024**, *12*, 2067. https://doi.org/10.3390/microorganisms12102067.
5. Fan, X.; Zhang, H.; Peng, Q.; Zheng, Y.; Shi, K.; Xia, X. Arsenic removal via the biomineralization of iron-oxidizing bacteria *Pseudarthrobacter* sp. Fe7. *Microorganisms* **2023**, *11*, 2860. https://doi.org/10.3390/microorganisms11122860.
6. Sventzouri, E.; Pispas, K.; Kournoutou, G.G.; Geroulia, M.; Giakoumatou, E.; Ali, S.S.; Kornaros, M. Evaluation of growth performance, biochemical composition, and polyhydroxyalkanoates production of four cyanobacterial species grown in cheese whey. *Microorganisms* **2025**, *13*, 1157. https://doi.org/10.3390/microorganisms13051157.
7. Quiñones-Cerna, C.; Castañeda-Aspajo, A.; Tirado-Gutierrez, M.; Salirrosas-Fernández, D.; Rodríguez-Soto, J.C.; Cruz-Monzón, J.A.; Hurtado-Butrón, F.; Ugarte-López, W.; Gutiérrez-Araujo, M.; Quezada-Alvarez, M.A.; et al. Efficacy of indigenous bacteria in the biodegradation of hydrocarbons isolated from agricultural soils in Huamachuco, Peru. *Microorganisms* **2024**, *12*, 1896. https://doi.org/10.3390/microorganisms12091896.
8. Afordoanyi, D.M.; Akosah, Y.A.; Shnakhova, L.; Saparmyradov, K.; Diabankana, R.G.C.; Validov, S. Biotechnological key genes of the *Rhodococcus erythropolis* MGMM8 genome: Genes for bioremediation, antibiotics, plant protection, and growth stimulation. *Microorganisms* **2024**, *12*, 88. https://doi.org/10.3390/microorganisms12010088.
9. Mqambalala, A.; Maleke, M.; Deysel, L.-M.; Osman, J.R.; Gomez-Arias, A.; Valverde, A.; Hernandez, J.C. First insight into the natural attenuation of emerging contaminants using a metagenomics approach from drinking water sources in the free state. *Microorganisms* **2025**, *13*, 2349. https://doi.org/10.3390/microorganisms13102349.
10. Chang, C.; Guo, Y.; Tang, K.; Hu, Y.; Xu, W.; Chen, W.; McLaughlin, N.; Wang, Z. Straw from different crop species recruits different communities of lignocellulose-degrading microorganisms in black soil. *Microorganisms* **2024**, *12*, 938. https://doi.org/10.3390/microorganisms12050938.
11. Mohd Noor, N.N.; Jeong, I.; Yoon, S.; Kim, K. Utilization of spent coffee grounds for bioelectricity generation in sediment microbial fuel cells. *Microorganisms* **2024**, *12*, 618. https://doi.org/10.3390/microorganisms12030618.
12. Mqambalala, A.; Maleke, M.; Osman, J.R.; Hernandez, J.C. Biodegradation of emerging contaminants controlled by biological and chemical factors. *Microorganisms* **2025**, *13*, 2354. https://doi.org/10.3390/microorganisms13102354.
13. Efremenko, E.; Stepanov, N.; Senko, O.; Aslanli, A.; Maslova, O.; Lyagin, I. Using fungi in artificial microbial consortia to solve bioremediation problems. *Microorganisms* **2024**, *12*, 470. https://doi.org/10.3390/microorganisms12030470.
